# GSK-3β inhibits autophagy and enhances radiosensitivity in non-small cell lung cancer

**DOI:** 10.1186/s13000-018-0708-x

**Published:** 2018-05-24

**Authors:** Jialin Ren, Tingting Liu, Yang Han, Qiongzi Wang, Yanzhi Chen, Guang Li, Lihong Jiang

**Affiliations:** 1 Department of Pathology, First Affiliated Hospital and College of Basic Medical Science, China Medical University, Shenyang, 110001 China; 2 Department of Surgery, First Affiliated Hospital, China Medical University, Shenyang, China; 3 Department of Radiotherapy, Fourth Affiliated Hospital, China Medical University, Shenyang, China; 4 Department of Radiotherapy, First Affiliated Hospital, China Medical University, Shenyang, China; 5 Department of Pathology, General Hospital of Liaohe Oilfield, Panjin, China

**Keywords:** X-rays, Non-small cell lung cancer, GSK-3β, Autophagy, Radiosensitivity

## Abstract

**Background:**

Radiotherapy is one of the most common and effective treatment methods for cancer, and improving the radiosensitivity of tumor tissues during the treatment process is vital. We report the mechanisms of glycogen synthase kinase 3 (GSK-3) β-regulated autophagy and the effects of autophagy on radiosensitivity in non-small cell lung cancer (NSCLC).

**Method:**

Immunohistochemical staining was performed to determine GSK-3β tissue expression in 89 NSCLC patients with follow-up data and the expression status of GSK-3β and autophagy in NSCLC tissues after X-ray radiotherapy. Western blots were used to quantitate changes in autophagy-related protein expression after A549 cells were treated with GSK-3β inhibitors and after H460 cells were transfected with GSK-3β mutants with different activities and X-ray irradiated. Clonogenic assays were used to measure the effect of autophagy on cellular proliferation.

**Results:**

GSK-3β expression positively correlated with NSCLC differentiation (*P* < 0.05), and GSK-3β negativity was associated with a better prognosis in 89 NSCLC patients. After X-ray irradiation, the expression levels of GSK-3β and p62 were decreased in NSCLC tissues, and the expression levels of the autophagy-related protein LC3 were increased. A549 and H460 cells were selected as representative GSK-3β-high and GSK-3β-low expression cell lines. After transfecting H460 cells with different GSK-3β mutants [wild type GSK-3β (GSK-3β-WT), constitutively active GSK-3β (GSK-3β-S9A), and catalytically inactive GSK-3β (GSK-3β-K85R)] and subjecting these cells to X-ray irradiation, AMPK and LC3 expression levels decreased, and p62 expression levels increased. These effects were particularly significant for the GSK-3β-S9A mutant. In A549 cells, after GSK-3β inhibition and X-ray irradiation, AMPK and LC3 protein expression levels increased. Moreover, when autophagy was inhibited, cell proliferation decreased.

**Conclusion:**

Our studies revealed that GSK-3β expression is associated with NSCLC differentiation, and patients with GSK-3β-negative tumors had a better prognosis. X-ray irradiation inhibited GSK-3β expression and promoted autophagy. Therefore, GSK-3β inhibits autophagy and enhances the radiosensitivity of NSCLC cells.

## Background

Glycogen synthase kinase 3 (GSK-3) β is a serine/threonine protein kinase, and its activity is regulated by the phosphorylation of specific sites. Tyr216 phosphorylation can enhance kinase activity, whereas Ser9 phosphorylation can inhibit kinase activity [1]. Recently, some studies have indicated that GSK-3β can regulate autophagy; however, others suggest that the regulatory effects of GSK-3β are not the same in different cancers [2–7].

Autophagy is a lysosome degradation process that plays a dynamic regulatory role in tumor occurrence [8]. Recent studies have shown that the autophagy status of cells affects the radiosensitivity of tumors [9]. In addition, these responses vary in different types of cells. Studies have shown that inhibiting autophagy in hepatocellular carcinoma, pancreatic cancer, prostate cancer, ovarian carcinoma, throat squamous cell carcinoma, and malignant gliomas can enhance radiosensitivity [10–15]. However, other studies show different results [16, 17], and the specific mechanisms associated with this process remain unclear.

Radiotherapy is one of the most common and effective treatment methods for cancer, and radioresistance due to hypoxia or drug resistance has marked effects on therapeutic efficacy [18]. Thus, improving the radiosensitivity of tumor tissues during the treatment process is vital [19]. Compared to small cell lung cancer, non-small cell lung cancer (NSCLC) accounts for the majority of lung cancer cases, and this malignancy is inherently radioresistant. Thus, investigating methods for increasing the radiosensitivity of NSCLC will be beneficial for targeted therapy regimens. As mentioned above, the role of GSK-3β in cancer, particularly NSCLC and radiosensitivity, is unclear.

In our study, we investigated the regulatory mechanisms of GSK-3β in autophagy and the effects of GSK-3β-regulated autophagy on radiosensitivity. We found that GSK-3β inhibits autophagy. When GSK-3β levels increased, autophagy levels decreased, and NSCLC radiosensitivity was enhanced. Consequently, the activation of kinases through phosphorylation plays an important role in GSK-3β-mediated autophagy inhibition.

## Methods

### Patients and specimens

We collected 89 tumor specimens from patients (67 males and 22 females) who underwent surgery at the First Affiliated Hospital of China Medical University between 2000 and 2004. All patients were diagnosed with squamous cell carcinoma, adenocarcinoma, or large cell carcinoma. In addition, all patients underwent curative surgical resection without prior chemotherapy or radiation therapy, and complete follow-up data were available.

According to the 2015 World Health Organization (WHO) classification of lung cancer, the samples included 45 squamous cell carcinomas, 41 adenocarcinomas, and three large cell carcinomas. A total of 16 tumors were well-differentiated, whereas 41 and 32 were moderately and poorly differentiated, respectively. Tumor staging was performed according to the TNM classification of the International Union Against Cancer (UICC). Sixty cases were pathological stage I or II, and 29 cases were stage III. The median age of the patients was 59 years (range: 33–76 years). This study was approved by the Institutional Review Board of China Medical University.

### NSCLC tissue X-ray exposure

Fresh tissues were isolated from 30 NSCLC patients (13 adenocarcinoma and 17 squamous cell carcinoma samples) and immediately cut into 4 mm × 4 mm × 1 mm blocks. The blocks were then cultured in Dulbecco’s modified Eagle’s medium (DMEM) (Gibco-BRL, Gaithersburg, MD) with 10% fetal calf serum at 37 °C. The tissues were irradiated (2 Gy) with X-rays (6 MV) at 37 °C using a linear accelerator (Primus, Siemens, Germany) as previously described [20]. Exposed tissues were cultured in DMEM for 5 h at 37 °C in a 5% CO_2_ incubator and then harvested.

### Immunohistochemistry

After irradiation with 2-Gy X-rays, samples were fixed in 10% neutral formalin, embedded in paraffin, and cut into 4-μm thick sections. Immunostaining was performed using the streptavidin-peroxidase method. The sections were incubated with GSK-3β (1:200, Santa Cruz, CA, USA), p-GSK-3β^Ser9^ (1:200, Bioss, China), p-GSK-3β^Tyr216^ (1:200, Bioss, China), LC3 (1:200, Wanleibio, China), AMPK (1:200, Wanleibio, China), and p62 (1:200, Wanleibio, China) primary antibodies at 4 °C overnight, followed by incubation with a biotinylated goat anti-rabbit IgG secondary antibody. The sections were incubated with horseradish peroxidase-conjugated streptavidin-biotin (Ultrasensitive; MaiXin, China), and diaminobenzidine (MaiXin) was used for coloration. Counterstaining was performed with Harris’s hematoxylin. The sections were dehydrated in graded concentrations of alcohol before mounting. The staining intensities were scored as 0 (no signal), 1 (weak), 2 (moderate), or 3 (high). Staining percentages were scored as 1 (1–25%), 2 (26–50%), 3 (51–75%), or 4 (76–100%). The scores of each tumor sample were multiplied to yield a final score of 0–12. Tumors with a score ≥ 3 were considered to be positive for GSK-3β expression. Tumor samples with scores between 1 and 2 were categorized as showing weak expression, whereas those with scores of 0 were defined as negative for GSK-3β expression.

### Cell culture

The human NSCLC cell lines A549, H460, H292, H1299, Calu1, and SK-MES-1 were obtained from the Shanghai Cell Bank (Shanghai, China). The HBE cell line was purchased from the American Type Culture Collection (ATCC; Manassas, VA, USA). Cells were cultured in RPMI 1640 (Invitrogen, Carlsbad, CA, USA) containing 10% fetal bovine serum (FBS; Invitrogen, Carlsbad, CA, USA) at 37 °C in 5% CO_2_.

### Western blotting

Total protein was extracted from cells with lysis buffer (Pierce, Rockford, IL, USA) and quantified with the Bradford method. A total of 50 μg of protein was separated using 10% SDS-PAGE and transferred onto PVDF membranes (Millipore, Billerica, MA, USA). The membranes were incubated overnight at 4 °C with primary antibodies against GSK-3β (1:1000, Santa Cruz Biotechnology), GAPDH (1:5000, Sigma, St. Louis, MO, USA), LC3 (1:1000, Wanleibio, China), AMPK (1:1000, Wanleibio, China), and p62 (1:1000, Wanleibio, China). After washing, the membranes were incubated with peroxidase-conjugated anti-mouse or anti-rabbit IgG (1:200, ZSGB-BIO, China) at 37 °C for 2 h. The protein bands were visualized using ECL (Pierce, Rockford, IL, USA), and images were captured using a bio-imaging system (DNR Bio-Imaging Systems, Jerusalem, Israel).

### Plasmid transfection

Wild type HA-GSK-3β-WT (GSK-3β-WT), constitutively active HA-GSK-3β-S9A (GSK-3β-S9A), and catalytically inactive HA-GSK-3β-K85R (GSK-3β-K85R) plasmids were gifts from Dr. Soo Young Lee (Division of Life and Pharmaceutical Sciences, Dept. of Bioinspired Science, Department of Life Science, Ewha Womans University). Lipofectamine 3000 transfection reagent was used for plasmid transfection (Invitrogen, USA) according to the manufacturer’s instructions.

### Colony formation assay

H460 cells were transfected with GSK-3β-WT, GSK-3β-S9A, GSK-3β-K85R, or negative control plasmids for 48 h. A549 cells were treated with the GSK-3 inhibitor SB216763 (Cell Signaling Technology, Danvers, MA, USA) for 24 h, and H460 cells were treated with rapamycin (KeyGEN Biotech, Nanjing, China) and 3-methyladenine (3-MA, KeyGEN Biotech) for 24 h. Cells were then irradiated with 2-Gy X-rays, transferred to three 6-cm cell culture dishes (1000 cells per dish) and incubated for 2 weeks. Then, the plates were washed with PBS and stained with Giemsa solution. Colonies with more than 50 cells were counted under a microscope. All experiments were performed in triplicate.

### Statistical analysis

SPSS 22.0 (SPSS, Chicago, IL, USA) was used for all analyses. A chi-square test was used to examine possible correlations between GSK-3β expression and clinicopathological factors. Kaplan-Meier survival analyses were performed for all 89 cases and compared using the log-rank test. Image data from the Western blots were compared by using the Mann-Whitney *U* test, and *P* < 0.05 was considered to indicate a statistically significant difference.

## Results

### GSK-3β expression in NSCLC is associated with differentiation and prognosis

**Fig. 1:**
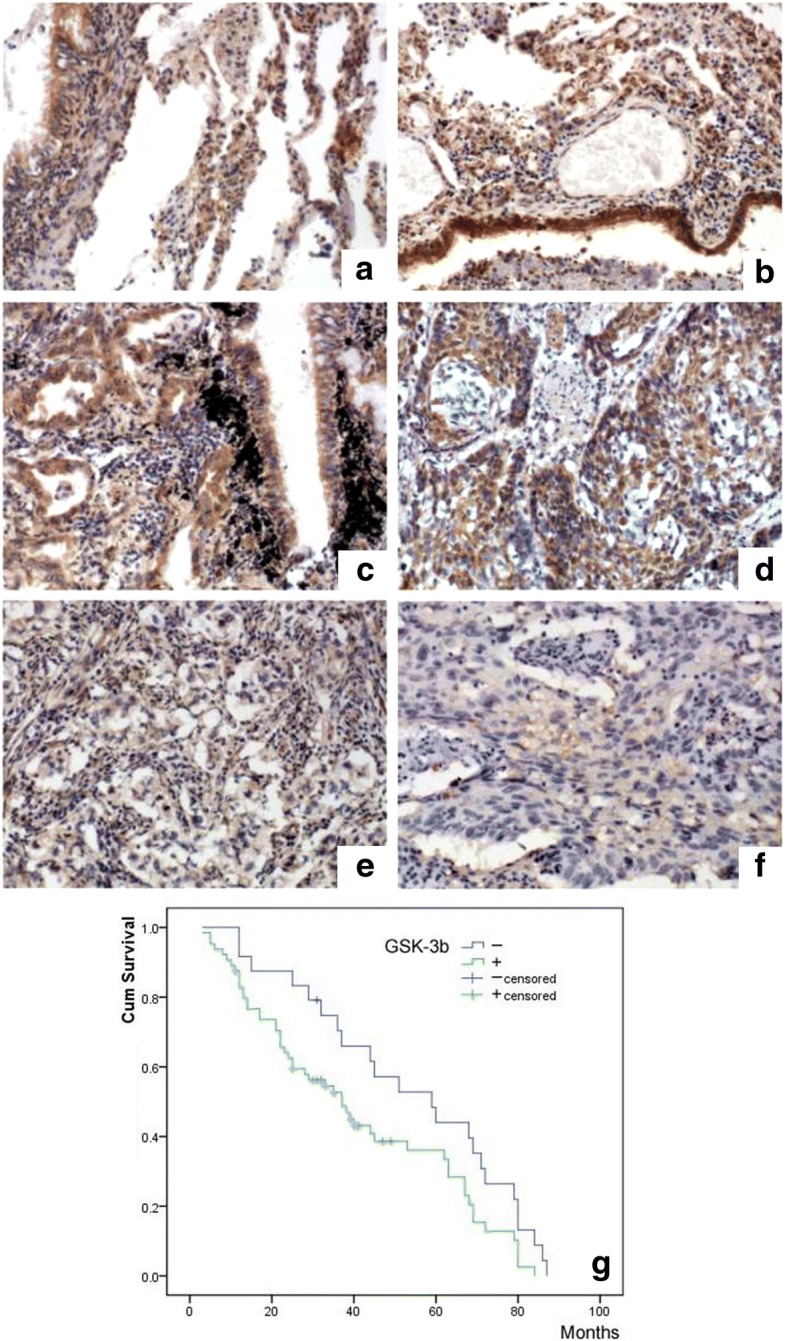
GSK-3β expression in NSCLC specimens. GSK-3β expression was strong (**a**, **b**) in the bronchioles and alveoli, positive in well-differentiated NSCLC (**c**, adenocarcinoma and **d**, squamous cell carcinoma), and weak or negative in poorly differentiated NSCLC (**e**, adenocarcinoma and **f**, squamous cell carcinoma). A Kaplan-Meier survival analysis showed that patients with negative GSK-3β expression had a significantly better prognosis than those with positive expression (**g**)

Immunohistochemical staining of 89 NSCLC tissue samples indicated that GSK-3β expression is intimately associated with the degree of differentiation. The positivity rate of highly differentiated cells (50.0%, 8/16) was significantly higher than that of moderately differentiated cells (19.51%, 8/41) and poorly differentiated cells (5.63%, 5/32) (*P* < 0.05) (Fig. 1). However, GSK-3β expression was not correlated with TNM tumor staging or lymph node metastasis (Table 1). A Kaplan-Meier analysis showed that patients with negative GSK-3β expression had a better prognosis than those with positive GSK-3β expression (Fig. 1).

**Table 1:** Correlation of GSK-3β expression with the clinicopathological features of 89 NSCLC patients

Clinicopathological factors	GSK-3β expression		
	–	+	*P* value
Sex			
Male	50	17	
Female	18	4	0.491
Age (years)			
< 59	36	6	
≥ 59	32	15	0.051
Histologic type			
SCC	31	14	
AC	34	7	
Others	3	0	0.192
Differentiation			
Well	8	8	
Moderate	33	8	
Poor	27	5	0.021
pTNM stage*			
I-II	45	15	
III	23	6	0.894

*TNM staging system of the International Union Against Cancer (UICC,2015)

### X-rays induce changes in GSK-3β, p-GSK-3β^Ser9^, and p-GSK-3β^Tyr216^ levels in NSCLC tissues

**Fig. 2:**
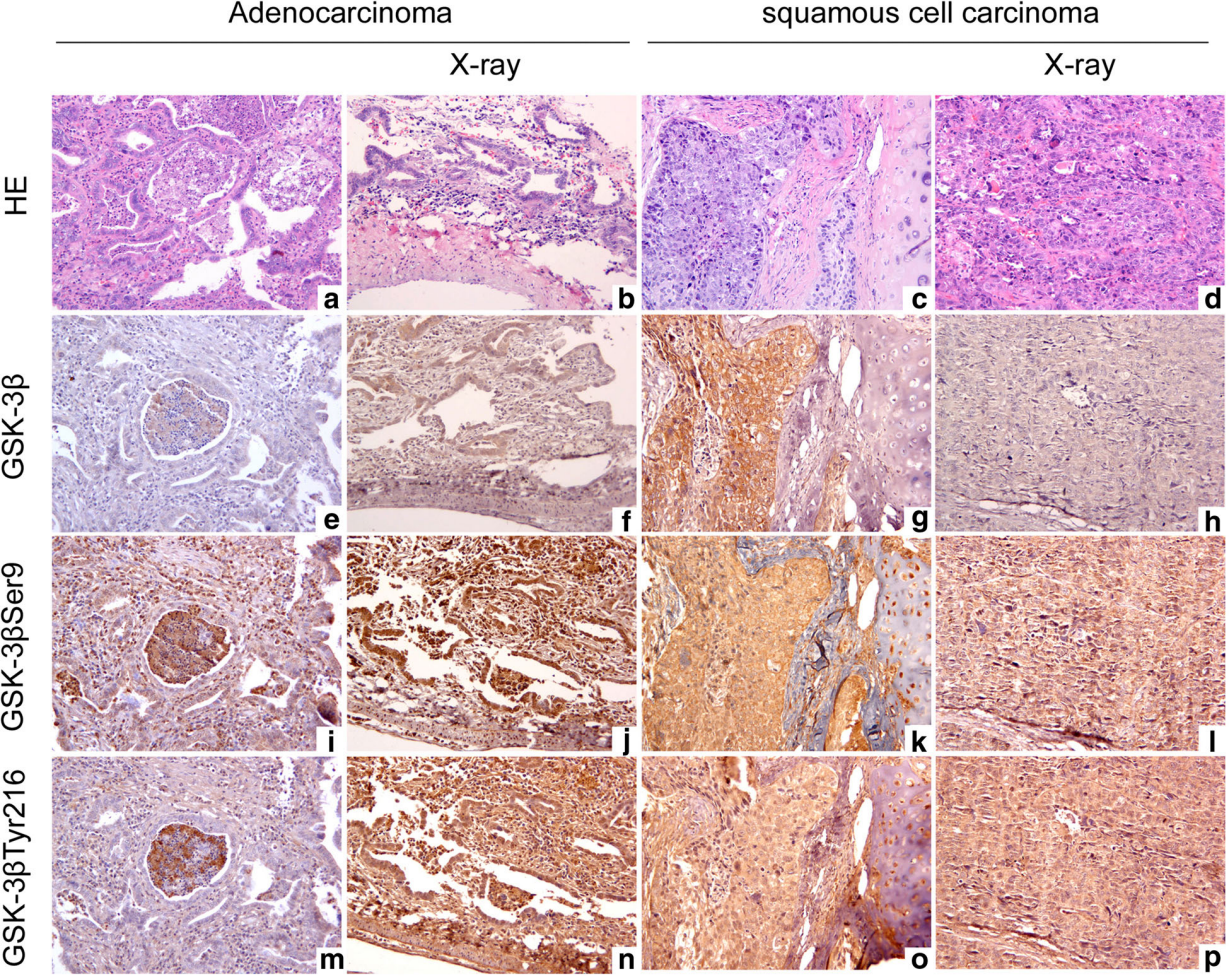
GSK-3β, p-GSK-3β^Ser9^, and p-GSK-3β^Tyr216^ expression in NSCLC tissues after X-ray irradiation. **a**, **e**, **i**, and M are the moderately differentiated adenocarcinoma tissues without X-ray irradiation, and **b**, **f**, **j**, and **n** are the tumor tissues that received 2-Gy X-ray irradiation. **a** and **b** are stained with HE (200×); **e** and **f** have immunohistochemical staining for GSK-3β; **i** and **j** have immunohistochemical staining for GSK-3β^Ser9^; and **m** and **n** have immunohistochemical staining for GSK-3β^Tyr216^. As shown in the figure, after 2-Gy X-ray irradiation of the adenocarcinoma samples, GSK-3β expression did not change significantly, but GSK-3β^Ser9^ and GSK-3β^Tyr216^ expression was significantly upregulated. **c**, **g**, **k**, and **o** are the poorly differentiated squamous cell carcinoma tissues without X-ray irradiation, and **d**, **h**, **l**, and **p** are the tumor tissues that received 2-Gy X-ray irradiation. **c** and **d** are stained with HE (200×); **g** and **h** have immunohistochemical staining for GSK-3β; **k** and **l** have immunohistochemical staining for GSK-3β^Ser9^; and **o** and **p** have immunohistochemical staining for GSK-3β^Tyr216^. As shown in the figure, after 2-Gy X-ray irradiation of the adenocarcinoma samples, GSK-3β and GSK-3β^Ser9^ expression was downregulated, whereas GSK-3β^Tyr216^ expression did not change significantly

After irradiating 30 NSCLC tissues with 2-Gy X-rays, we found no significant changes in GSK-3β protein expression levels in 12 patient samples (moderately differentiated adenocarcinoma), but p-GSK-3β^Ser9^ and p-GSK-3β^Tyr216^ levels were significantly increased (Fig. 2). In the other 10 patient samples (poorly differentiated squamous cell carcinoma), decreased GSK-3β and p-GSK-3β^Ser9^ protein expression levels were observed, but no significant changes in p-GSK-3β^Tyr216^ levels were identified (Fig. 2).

### X-rays induce changes in autophagy makers in NSCLC tissues

**Fig. 3:**
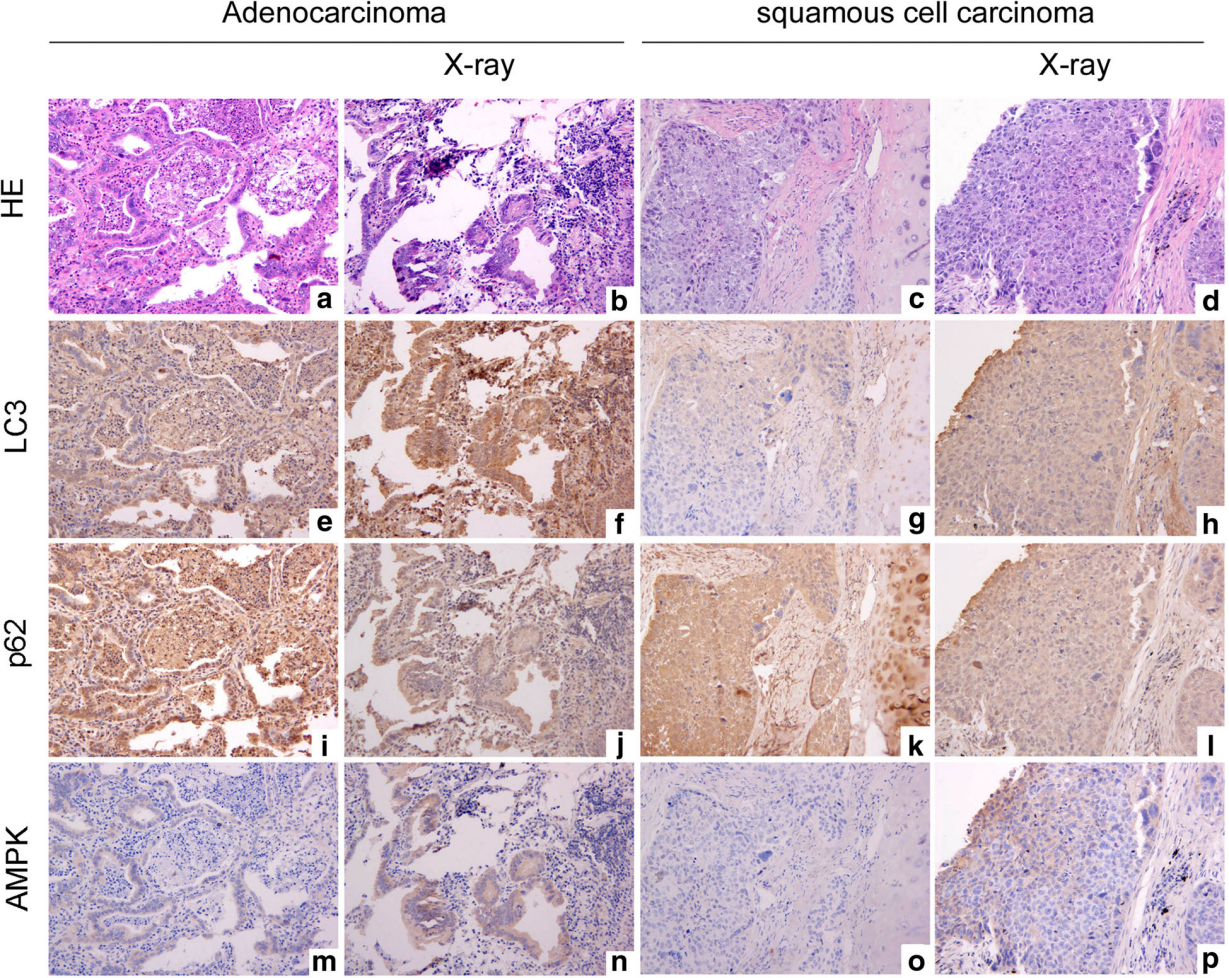
LC3, P62 and AMPK expression in NSCLC tissues after X-ray irradiation. After 2-Gy X-ray irradiation of the adenocarcinoma samples (moderately differentiated, **a** and **b** are stained with HE, 200×), LC3 expression was significantly upregulated (**e** is the control, and **f** indicates LC3 upregulation in the adenocarcinoma samples after X-ray irradiation); p62 expression was downregulated (**i** is the control, and **j** indicates p62 downregulation in the adenocarcinoma samples after X-ray irradiation); and AMPK expression was upregulated (**m** is the control, and **n** indicates AMPK upregulation in the adenocarcinoma samples after X-ray irradiation). After 2-Gy X-ray irradiation of the squamous cell carcinoma samples (poorly differentiated, **c** and **d** are stained with HE, 200×), LC3 expression was significantly upregulated (**g** is the control, and **h** indicates LC3 upregulation in the squamous cell carcinoma samples after X-ray irradiation); p62 expression was downregulated (**k** is the control, and **l** indicates p62 downregulation in the squamous cell carcinoma samples after X-ray irradiation); and AMPK expression was slightly upregulated (**o** is the control, and **p** indicates slight AMPK upregulation in the squamous cell carcinoma samples after X-ray irradiation)

After irradiating 30 NSCLC tissue specimens with 2-Gy X-rays, we found that LC3 protein expression levels were significantly increased in 26 samples (11 adenocarcinoma, 15 squamous cell carcinoma, 18 moderately differentiated and 8 highly differentiated samples); in addition, p62 protein expression levels were decreased, and AMPK protein expression levels were increased (Fig. 3).

### Effects of GSK-3β on X-ray-induced changes in autophagy

**Fig. 4:**
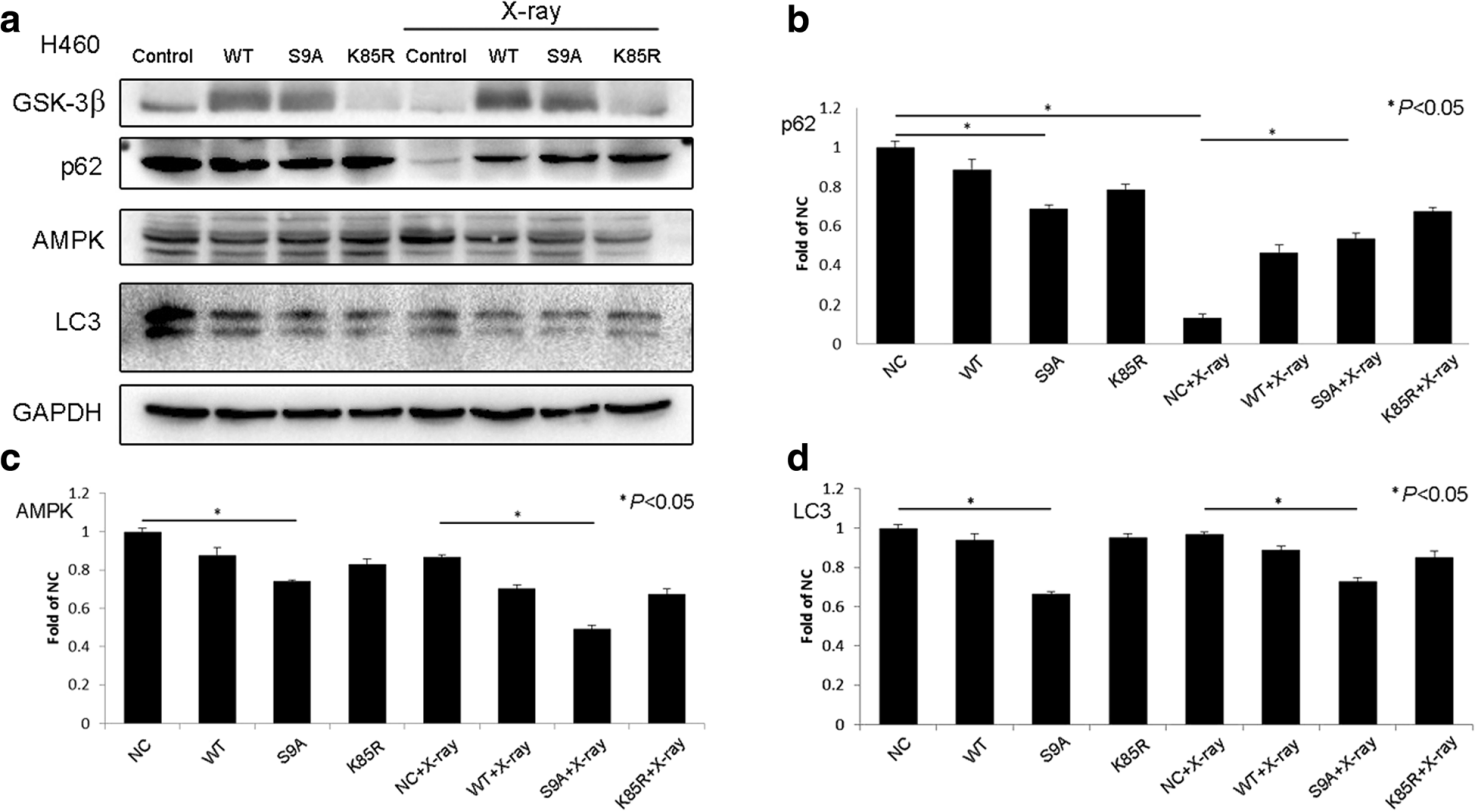
GSK-3β inhibited cellular autophagy. Western blotting showed that (**a**) after X-ray irradiation alone, GSK-3β and p62 levels decreased, whereas AMPK and LC3 levels did not change significantly in H460 cells. After transfection with GSK-3β with different activity levels, p62, AMPK, and LC3 levels decreased. After transfection with GSK-3β with different activity levels and X-ray irradiation, p62 levels increased, whereas AMPK and LC3 levels decreased. These changes were more significant for transfection with the active GSK-3β-S9A mutant than for transfection with the GSK-3β-WT or inactivated GSK-3β-K85R mutant (**b**, **c**, **d**). *P < 0.05

**Fig. 5:**
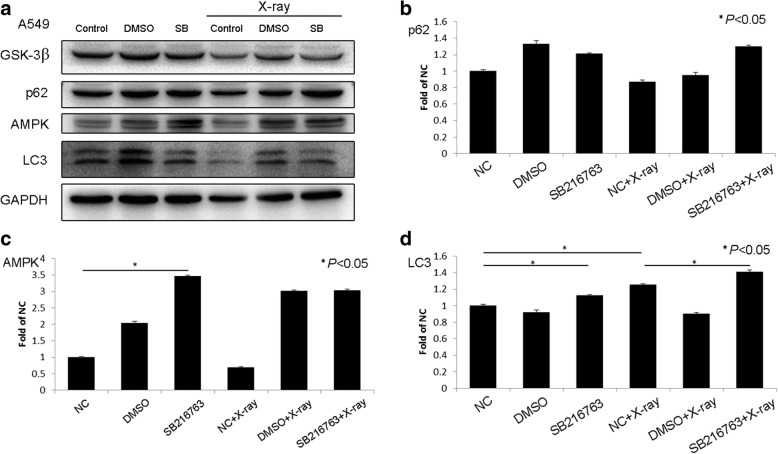
GSK-3β inhibition promoted cellular autophagy. Western blotting showed that (**a**) after X-ray irradiation alone, GSK-3β levels decreased; p62 and AMPK levels did not change significantly; and LC3 levels increased in A549 cells. After treatment with the GSK-3 inhibitor SB216763, p62 levels did not change significantly, whereas AMPK and LC3 levels increased. After treatment with the GSK-3β inhibitor SB216763 and X-ray irradiation, p62 and AMPK levels did not change significantly, whereas LC3 levels increased (**b**, **c**, **d**). *P < 0.05

To show that GSK-3β can affect the X-ray-induced expression of autophagy markers, we utilized H460 cells, which express GSK-3β, for transfection, and we inhibited GSK-3β in A549 cells. The treated cells were irradiated with 2-Gy X-rays. The results showed that after transfection with GSK-3β-WT and constitutively active GSK-3β-S9A, LC3 protein expression levels were decreased, and autophagy was inhibited. Transfection with the catalytically inactive GSK-3β-K85R plasmid did not significantly change autophagy levels (Fig. 4). Conversely, GSK-3β inhibition increased AMPK and LC3 protein expression levels, and these changes are indicative of autophagy. After X-ray irradiation only, GSK-3β and p62 protein levels decreased, and LC3 protein levels increased; these findings suggest that autophagy was promoted (Fig. 5). After GSK-3β transfection and X-ray irradiation, AMPK and LC3 protein expression levels decreased, and p62 protein expression levels increased. Of the GSK-3β mutants, GSK-3β-S9A yielded greater effects than GSK-3β-WT and the catalytically inactive GSK-3β-K85R. Conversely, GSK-3β inhibition followed by X-ray irradiation upregulated AMPK and LC3 protein expression, suggesting that autophagy was promoted.

### Effects of autophagy on X-ray-induced cell growth

**Fig. 6:**
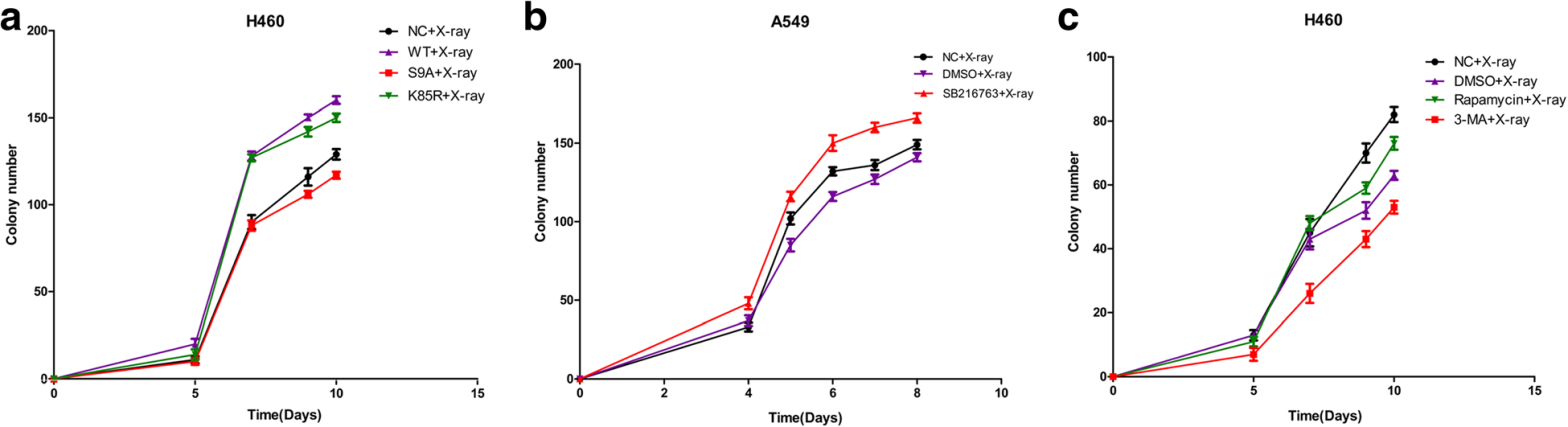
Clonogenic ability of cells after treatment and X-ray irradiation. Colony formation in H460 cells transfected with GSK-3β with different activity levels [wild type GSK-3β (GSK-3β-WT), constitutively active GSK-3β-S9A, and catalytically inactive GSK-3β-K85R)] and X-ray irradiation (**a**). After transfection with GSK-3β with different activity levels, the clone-forming ability of activated GSK-3β-S9A was reduced, and cell viability was decreased. Colony formation in A549 cells after GSK-3β inhibition (SB216763) and X-ray irradiation (**b**). After inhibiting GSK-3β activity, the clone-forming ability and cell viability increased. Colony formation in H460 cells after inhibiting or promoting autophagy (cells were treated with the autophagy inducer rapamycin and the autophagy inhibitor 3-MA) and X-ray irradiation (**c**). After inhibiting autophagy with 3-MA, the reduction in clone-forming ability was more significant, and cell viability was decreased

To examine the effects of autophagy on X-ray-induced cell growth, we determined the clonogenic ability of cells after treatment and X-ray irradiation. The results showed that after transfection with GSK-3β-S9A, clonogenic ability and cell survival were decreased. After GSK-3β inhibition, clonogenic ability and cell survival increased. We also used 3-MA to inhibit autophagy and rapamycin to increase autophagy. The results showed that upon X-ray induction, inhibiting autophagy decreased clonogenic ability and cell survival (Fig. 6).

## Discussion

Our experimental results showed that GSK-3β is intimately associated with the degree of differentiation in NSCLC but is not correlated with tumor TNM staging or lymph node metastasis. Patients with negative GSK-3β expression had a better prognosis than those with positive GSK-3β expression. GSK-3β can be expressed as wild type, constitutively active, and catalytically inactive forms. All of these GSK-3β forms were assessed in the clinical samples. Consequently, our results might have been due to the inability of the GSK-3β antibodies used in our study to distinguish between the constitutively active and catalytically inactive forms of GSK-3β. X-ray irradiation resulted in the downregulation of GSK-3β and the upregulation of p-GSK-3β^Ser9^ and p-GSK-3β^Tyr216^ in NSCLC tissues. GSK-3β is a type of serine/threonine protein kinase, and its activity is regulated by the phosphorylation of specific sites. Tyr216 phosphorylation can increase kinase activity, whereas Ser9 phosphorylation can inhibit kinase activity [1]. Therefore, we hypothesized that GSK-3β activity is associated with radiosensitivity in NSCLC tissue and that kinase activation through phosphorylation plays an important role. Previous studies have shown that X-ray irradiation can change the activation status of GSK-3β to regulate tissue radiosensitivity, but the specific effector mechanisms remain unclear [21, 22]. One study has found that the radioresistance was lower in human non-small cell lung cancer (NSCLC) cell lines when the miR-21 level is lower. GSK-3β regulated radiosensitivity through miR-21, high miR-21 levels has a negative correlation with low GSK-3β levels in different human tumors (pheochromocytoma/paraganglioma, kidney tumor and testicular germ cell tumors) [23]. We think the differential results in these studies might be due to the different cell lines and the specific pathway to radioresistance was also different. In the next study, we will increase the NSCLC cell lines and the number of NSCLC specimens.

Our study showed that X-ray irradiation can increase autophagy levels in NSCLC tissues. Cancer cells are thought to survive due to autophagy, and this effect presents as radioresistance [24, 25]. However, autophagy can have opposing effects on cell survival. In different stages of tumor growth, autophagy plays completely different roles. In the early phases of tumor growth, inhibiting autophagy can increase anabolism and cancer cell proliferation and decrease protein degradation. At this early stage, autophagy plays a tumor suppressive role. As the tumor progresses, and particularly when there is an insufficient number of blood vessels to supply nutrients to the tumor, tumor cells can overcome nutrient deprivation and hypoxia and survive by upregulating autophagy. This process can also remove mitochondria from cells to protect cells from radiation damage and support continuous tumor proliferation. In different types of tumors, the functions of autophagy are also different. Some studies have found that autophagy can inhibit tumor cell proliferation [26–29]. However, other studies have found that this process can increase tumor cell death [30–38]. Studies have shown that the autophagy status of cells can affect radiosensitivity in tumors [9]. Our study shows that X-rays induce changes in GSK-3β, p-GSK-3β^Ser9^, and p-GSK-3β^Tyr216^ levels in NSCLC tissues, and we hypothesized that GSK-3β may regulate autophagy in NSCLC to alter radiosensitivity.

We also showed that GSK-3β can affect X-ray-induced autophagy, as increased GSK-3β expression inhibited autophagy, and this effect was more significant with the constitutively active variant. In addition, GSK-3β inhibition resulted in increased autophagy. Similar studies on skin squamous cell carcinoma [2], breast carcinoma [3], prostate adenocarcinoma [4], and pancreatic carcinoma [5] all support this result. However, some studies have demonstrated results completely opposite to those of our study regarding how GSK-3β phosphorylation affects autophagy [6, 7]. These differences might be due to the type of cancer studied and the nutritional status of the cells. The exact mechanisms associated with these differences require further investigation. The specific pathway through which GSK-3β regulates autophagy also requires further examination.

Additionally, we examined the effects of GSK-3β-regulated autophagy on radiosensitivity. We found that GSK-3β inhibited cell proliferation, and this effect was more pronounced with the constitutively active variant. Inhibiting autophagy also inhibited cell proliferation, which could lead to increased radiosensitivity. The results of previous studies that used different types of tumors, including hepatocellular carcinoma [10], pancreatic cancer [11], prostate cancer [12], ovarian carcinoma [13], throat squamous cell carcinoma [14], and malignant gliomas [15], also support our findings. However, in breast adenocarcinoma, promoting autophagy can increase radiosensitivity [16]. This result might be due to vitamin D3 activity in breast adenocarcinoma [17]. The differential results reported in these studies might be due to the type of tumor and other related factors. Thus, the specific associated mechanisms remain unclear.

## Conclusions

In summary, our study showed that GSK-3β can inhibit autophagy in NSCLC and that phosphorylation levels and sites play crucial roles in this process. Moreover, GSK-3β inhibited autophagy to increase radiosensitivity in NSCLC. These results provide new insight into NSCLC radiosensitivity and corresponding clinical treatments.

## Abbreviations

NSCLC: Non-small cell lung cancer
UICC: International Union Against Cancer
WHO: World Health Organization
